# Chronic lymphocytic leukaemia/small lymphocytic lymphoma treatment with rituximab and high‐dose methylprednisolone, revisited

**DOI:** 10.1002/cam4.4374

**Published:** 2021-11-16

**Authors:** Ana Vagos Mata, Eduardo Espada, Daniela Alves, Blanca Polo, Maria João Costa, Conceição Lopes, João F. Lacerda, João Raposo

**Affiliations:** ^1^ Hematology and Bone Marrow Transplant Department Hospital de Santa Maria Centro Hospitalar Universitário Lisboa Norte Lisbon Portugal; ^2^ Instituto de Medicina Molecular João Lobo Antunes Faculdade de Medicina Universidade de Lisboa Lisbon Portugal; ^3^ Faculdade de Medicina Universidade de Lisboa Lisbon Portugal

**Keywords:** auto‐immune haemolytic anaemia, chronic lymphocytic leukaemia, high‐dose methylprednisolone, rituximab

## Abstract

High‐dose methylprednisolone plus rituximab (R‐HDMP) is a useful treatment in chronic lymphocytic leukaemia/small lymphocytic lymphoma (CLL/SLL) patients unfit for chemo‐immunotherapy and has proven its utility on the treatment of CLL/SLL complicated by auto‐immune cytopenias. We performed a retrospective, single‐centre study, of CLL/SLL patients treated with R‐HDMP for 9 years. Thirty‐nine patients were included, median age at time of treatment was 77 years. Most patients had stage Rai III/IV and Binet C disease. Twenty‐eight patients had relapsed/refractory disease at time of treatment with a median of 1 previous line of therapy; 53.8% had prior exposure to fludarabine and 25% to rituximab. Grade 3–4 neutropenia and thrombocytopenia were recorded in 10.2% and 17.9% patients, respectively. While on treatment, 51.3% had documented infectious complications, but no other non‐haematological toxicities grades 3–4 were identified. Overall response rate was 64%. Median overall survival and progression‐free survival were 24 and 13 months, respectively. Twenty four patients relapsed and 16 received another line of treatment after R‐HDMP, with median time to next treatment of 13.5 months. Thirteen out of the 24 patients improved performance status and were subsequently considered fit for chemo‐immunotherapy. R‐HDMP is a valuable option for elderly and frail patients, with low risk of severe myelotoxicity and other severe adverse events. It was shown to work as a bridge to other lines of treatment, including chemo‐immunotherapy.

## Graphical abstract

High dose methylprednisolone plus rituximab (R‐HDMP) is a useful treatment in chronic lymphocytic leukaemia/small lymphocytic lymphoma patients unfit for chemo‐immunotherapy. We present a cohort of patients with median age of 77 years, most with stage Rai III/IV and Binet C disease. Overall response rate was 64% and median overall survival and progression‐free survival were 24 and 13 months, respectively, with an acceptable toxicity profile, highlighting R‐HDMP utility for elderly and frail patients.

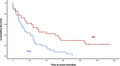

## 1 INTRODUCTION

Chronic lymphocytic leukaemia/small lymphocytic lymphoma (CLL/SLL), an indolent malignancy of mature B cells that predominantly affects men, is the most frequent haematologic malignancy in developed countries. Its incidence increases with age and roughly half of the patients will never need treatment.^1^ According to the International Workshop on Chronic Lymphocytic Leukaemia (iwCLL) guidelines, treatment is warranted when active disease criteria are met, which include steroid‐refractory autoimmune cytopenias.^2^

CLL patients have a 5%–10% cumulative risk of developing autoimmune phenomena, which bear no prognostic impact and can happen in any stage of disease. Actually, patients with CLL and immune cytopenias have a better prognosis than those with cytopenias due to marrow infiltration.^3^ Some authors state that auto‐immune cytopenias may occur in up to 25% of cases, mostly associated with high risk features, such as del(17p), del(11q), and unmutated immunoglobulin heavy‐chain variable region (IgHV) gene.^4^ Cytopenias are the most frequent autoimmune complications in CLL. Autoimmune haemolytic anaemia (AIHA) is by far the most frequent autoimmune cytopenia, while immune thrombocytopenia, pure red cell aplasia and immune agranulocytopenia are less frequent.^5^, ^6^ There are at least three mechanisms under the appearance of autoimmune cytopenias: malignant lymphocytes may be responsible for aberrant antigen presentation (Rh antigen, for example); CLL cells may also produce inhibitory cytokines that impair tolerance mechanisms; there may also be an imbalance between T‐cells subsets, favouring the appearance of auto‐reactive B cells against erythrocytes and platelets.^3^, ^4^ It is extremely important to understand if the cytopenias are a result of autoimmune phenomena, marrow infiltration, bleeding loss, renal disease or vitamin/iron deficits. The correct laboratory work‐up is fundamental to correctly diagnose the cause of the cytopenia. Importantly, a positive direct anti‐globulin test (DAT) is not diagnostic of AIHA if the other laboratory features are not present; on the other hand, AIHA may occur even with negative DAT, either because of low affinity or low titers of antibodies.^4^ When isolated, autoimmune cytopenias can be treated with steroids; when steroid‐refractory or in association with progressive CLL (termed complex autoimmune cytopenia), chemo‐immunotherapy protocols are recommended.^7^

CLL treatment protocols have evolved significantly over the last 20 years. There is currently a consensus about first‐line therapy for fit young patients, with some variations according to TP53 and IgHV mutational status. There are also interesting alternatives for those who have some comorbidities, considered “slow go*”* patients.^8^ A bigger challenge is to decide how to treat older and very frail patients, either in first‐line or in the relapsed/refractory setting. Most of these patients have multiple comorbidities and more than half suffer from inadequate polypharmacy, leading to low adherence to new therapies and dangerous drug interactions.^9^ This concern is particularly relevant in long‐term fixed therapy and therapy until progression, as opposed to short‐fixed duration therapy, despite recent work showing safety and efficacy of new drugs on elderly patients.^10^, ^11^ The last position statement from the Society of Geriatric Oncology highlights the importance of assessing elderly patients according to their physical and cognitive capacity, as well as to their ability of performing activities of daily living. The authors highlight that no frailty score has been prospectively validated in CLL, and that such elderly patients are rarely included in clinical trials, making it extremely difficult to make the right decision about these patients. Such an intricate evaluation is necessary to choose a treatment protocol that is feasible for each patient, meaning that it does not lead to discontinuation, dose reduction, or delay in treatment protocol. They also refer that the economic impact of new therapies in elderly patients should not be forgotten, suggesting that further evaluation of the health economic impact of the new drugs in elderly patients should be performed, in order to prioritise treatments.^12^

A high‐dose methylprednisolone (HDMP) protocol for CLL was first published by Thornton et al in 1999.^13^ Since then, many authors have published their experience with rituximab plus high‐dose steroid therapy, either dexamethasone or methylprednisolone (R‐HDMP). These protocols have small variations regarding rituximab dose, steroid dose, and number of days of steroid administration, and have shown interesting results, especially in the relapsed/refractory setting, with overall response rates varying from 28% to 75%.^14^, ^15^, ^16^ Of note, the median patient age in these trials was 66–73 years old. These protocols have also showed efficacy in patients with unfavourable cytogenetics and TP53 mutation in the pre‐Bruton's tyrosine kinase inhibitor (BTKi) era.^17^

In the current study, we present our results of R‐HDMP in an elderly, frail and mostly pre‐treated CLL patient population.

## 2 PATIENTS AND METHODS

We retrospectively analysed 39 CLL/SLL patients treated with R‐HDMP from 2009 to 2018 in a Haematology and Bone Marrow Transplant Department of a tertiary hospital. CLL diagnosis was performed according with iwCLL guidelines: sustained lymphocytosis (>5000 lymphocytes/µL for more than 3 months) with immunophenotype positive for CD19, CD5, CD23 and CD20^dim^, with ƙ or ʎ light chain restriction. Bone marrow evaluation was performed in 82% of patients at diagnosis. Molecular characterisation of the disease was performed through *fluorescence in situ hybridisation* searching for del(13q), trisomy of chromosome 12, del(11q) and del(17p). Searching for TP53 mutations was not routinely performed by then, so that information was not included. Mutational status of immunoglobulin variable heavy chain (IgHV) gene was assessed in most patients. Data regarding the presence and degree of anaemia, thrombocytopenia, B symptoms, degree of lymphocytosis, level of lactate dehydrogenase (LDH), level of β2‐microglobulin and patients’ comorbidities at the time of treatment were collected from clinical registries. Cummulative Ilness Rating Scale (CIRS) was calculated considering patient's comorbidities. CLL diagnosis and medical conditions thought to be complications of CLL were not included as part of the total CIRS score.^18^

The R‐HDMP treatment protocol consisted of rituximab 500 mg/m^2^ once weekly, IV, for 4 consecutive weeks, along with methylprednisolone 1 g/day, IV, 3 consecutive days every week for 4 consecutive weeks. Physician criteria for choosing this protocol over other therapies were older age, multiple comorbidities, lack of adherence to oral therapy or presence of auto‐immune haemolytic anaemia.

Antimicrobial prophylaxis was given at the discretion of the investigator: all patients received prophylaxis with sulfamethoxazole‐trimethoprim; acyclovir and fluconazole prophylaxis were given to 30% and 25%, respectively. Granulocyte colony‐stimulating factor was only given on the event of grade IV neutropenia (less than 500 neutrophils/µl). Assessment of prognostic factors, imaging studies and routine laboratory tests were performed according to standard practice. Response to treatment was assessed according to iwCLL criteria, 3 months after end of treatment.

This study was conducted according to Helsinki declaration principles and under the approval of local Ethics Committee. All patients signed an informed consent before treatment.

### 2.1 Statistical analysis

Descriptive statistics were used to analyse patients’ characteristics, disease profile, response to treatment and toxicity profile. Differences between patients’ characteristics in frontline and relapsed/refractory setting were analysed through Pearson chi‐square and Mann–Whitney tests, for qualitative and quantitative variables, respectively. Time intervals were measured from the first day of treatment until progressive disease (PD), additional CLL treatment or death. Survival analysis was performed using the Kaplan–Meier method; differences in survival according to patients’ or disease characteristics were compared by log‐rank test. Independent predictors of progression or mortality were assessed by Cox regression univariate analysis. Multivariate analysis was not performed, given the absence of multiple significant prognostic predictors in univariate analysis. *p* < 0.05 was considered to be statistically significant. Statistical analysis was done using IBM® SPSS® Statistics version 25.

## 3 RESULTS

### 3.1 Patient characteristics

Thirty‐nine patients were included, 27 of whom were male (69.2%), median age at time of treatment was 77 years (inter‐quartile range [IQR]: 71–80 years). Most patients displayed highly unfavourable clinical and cytogenetic characteristics (Table 1). More than half (61.5%) had high‐risk (Rai III/IV and Binet C) disease. Median lymphocytosis was 45,390 cel/µL, with 18% patients with lymphocytosis between 50,000 and 100,000 cel/µl and 31% with lymphocytosis above 100,000cel/µl. At the time of treatment, 53.8% had multiple adenopathy, 18% had B symptoms, 36% had recurrent infections and 70% had cytopenias (61% with anaemia and 41% with thrombocytopenia). Haemoglobin (Hb) levels were below 10 g/dl in 53.8% of patients and below 8 g/dl in 18% of patients. Platelet count below 50,000 cel/µl in 20.5% of patients. Fourteen patients (36%) had auto‐immune haemolytic anaemia. LDH was evaluated in all patients, with median LDH of 530 U/L, IQR of 401–826 U/L (upper limit of normal of local laboratory: 250 U/L). β2‐microglobulin was evaluated in 28.2% of patients at time of treatment with R‐HDMP, with all patients having a value above the reference range (0.8–3.0 mg/L). Median β2‐microglobulin was 3.86 mg/L. Albumin was evaluated in 62% of patients: median albumin level was 3.85 g/dl (3.4–5.4 g/dl), with 32% of patients with albumin below de reference level. C‐reactive protein (CRP) was evaluated in 46% of patients at time of treatment, with half of patients having negative CRP. Median CRP of 0.7 mg/dl (reference range <0.5 mg/dl). Median CIRS (Cumulative Illness Rating Scale) was 5, with 41% of patients having CIRS ≥6. Twelve patients (30.8%) had chronic kidney disease KDIGO stage 3a or higher (creatinine clearance [CrCl] <60 ml/min/1.73 m^2^), 56% of patients had arterial hypertension, 10.3% had type II diabetes mellitus, 12.8% had dyslipidaemia, 17.9% had valvular or ischaemic cardiomyopathy, 10.3% had chronic obstructive pulmonary disease and 15.4% had psychiatric disorders. IgHV mutational status was assessed in 23 patients, 17 of whom (73.9%) had unmutated disease. FISH cytogenetic profile was studied in 35 patients, 11.4% of whom had del(17p), 22.9% del(11q), 28.6% had trisomy of chromosome 12 and 57.1% had del(13q). Twenty‐eight patients (71.8%) had relapsed/refractory (R/R) disease at time of treatment with a median of one previous line of therapy and a maximum of four. Patients’ characteristics were equally distributed between frontline and relapsed/refractory setting, except for the presence of recurrent infections, which were more common on the relapsed/refractory setting (*p* = 0.02) and for the median lymphocytosis, higher in frontline setting than in relapsed/refractory setting (median lymphocytosis of 108,740 cel/µl and 38,3400 cel/µl, respectively, *p* = 0.02). Most patients (53.8%) had prior exposure to fludarabine and 25% had prior exposure to rituximab.

**TABLE 1:** Baseline characteristics; quantitative variables expressed as median/range and qualitative variables expresses as number/%

Baseline characteristics	*N* = 39
Gender	
Male	27 (69.2)
Female	12 (30.8)
Age	77 (70–81)
RAI	
0	1 (2.6)
1	3 (7.7)
2	8 (20.5)
3	19 (48.7)
4	5 (12.8)
BINET	
A	8 (20.5)
B	5 (18.8)
C	26 (61.5)
CIRS	
Median (min–max)	5 (2–13)
ClCr (ml/min/1.73 m^2^)	
≥60	27 (69.2%)
<60	12 (30.8%)
Cytogenetic profile (FISH)	*N* = 35
del(17p)	4 (11.4%)
del(11q)	8 (22.9%)
triss 12	10 (28.6%)
del(13q)	20 (57.1%)
Normal	6 (17.14%)
IgHV mutational status	*N* = 23
Mutated	6 (26.1%)
Unmutated	17 (73.9%)
Relapsed/refractory disease	*N* = 39
Yes	28 (71.8%)
No	11 (28.2%)
Previous lines of therapy	*N* = 39
Median (min‐max)	1 (0–4)
Prior exposure to fludarabin	*N* = 39
Yes	21 (53.8%)
No	18 (42.6%)

### 3.2 Treatment plan

The scheduled treatment protocol was completed in 25 patients (64.1%), with five other (12.9%) completing at least three cycles. Three patients (7.7%) received more than four cycles (median of cycles: 4; range 1–8). Of the remaining 6 patients, 2 patients died during treatment, 2 patients completed 1 week of treatment with PD and data are missing regarding the number of cycles performed on two other patients. Nevertheless, on these last two, evaluation response was performed 3 months after the end of treatment. All patients received treatment in an outpatient setting.

### 3.3 Toxicity

Grade 3–4 neutropenia and thrombocytopenia were recorded in 4 (10.2%) and 7 (17.9%) patients, respectively. Twenty patients (51.3%) had documented infectious complications while on treatment (grade 3–4: 60%), but no other grade 3–4 non‐haematological toxicities were identified, namely metabolic decompensation, tumour lysis syndrome and steroid induced psychosis.

### 3.4 Response to therapy

Of the 35 patients with assessed response, overall response rate (ORR) was 64% (complete response [CR] = 17.9%, CR with incomplete bone marrow recovery [CRi] = 5.1%, partial response [PR] = 41%); 5.1% and 20.5% of patients had stable disease (SD) and PD, respectively. Of the four patients not evaluated for response, two died during treatment, one died 2 months after finishing treatment and one patient did not complete response assessment exams for reasons unrelated to medical condition (Table 2). Patients treated in relapsed setting had similar ORR compared with patients in frontline (ORR 64.4% vs ORR 66.6%, respectively; *p* = 0.741). Patients with AIHA had better ORR compared with the remaining patients, albeit without statistical significance (ORR 85.7% vs. ORR 54.5%, *p* = 0.118). Of the four patients with del(17p), three achieved a CR after four cycles of R‐HDMP (ORR 75%) and one patient had SD. Of the 35 patients with assessed response, median time to normal lymphocyte (<4500 cel/µl) and haemoglobin (Hb >12 g/dl) count was 16 days (IQR 13–36 days) and 23 days (14–51 days), respectively. Of the 16 patients with thrombocytopenia, only four achieved normal platelet count (minimum of 15 days and maximum of 62 days). Patients treated in relapsed setting had similar median time to haemoglobin and lymphocyte recovery, compared with frontline setting (*p* = 0.17 and *p *= 0.78, respectively).

**TABLE 2:** Response to treatment; qualitative variables expresses as number/%

	*N* = 39
Overall response rate	64
Complete remission	7 (17.9)
Complete remission with incomplete marrow recovery	2 (5.1)
Partial remission	16 (41)
Stable disease	2 (5.1)
Progressive disease	8 (20.5)
Not assessed	4 (10.3)

### 3.5 Follow‐up

**FIGURE 1:**
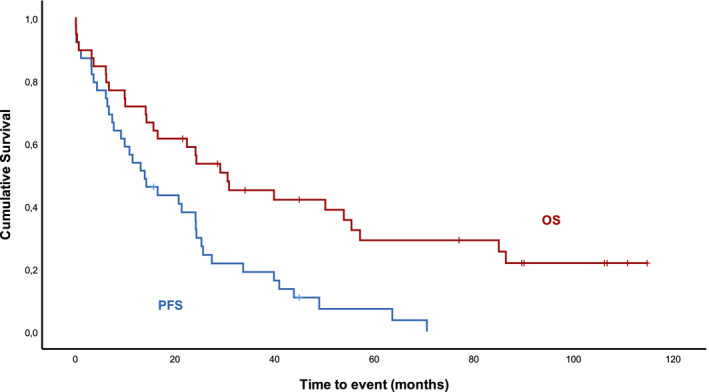
Overall (OS) and progression free survival (PFS) after R‐HDMP

**FIGURE 2:**
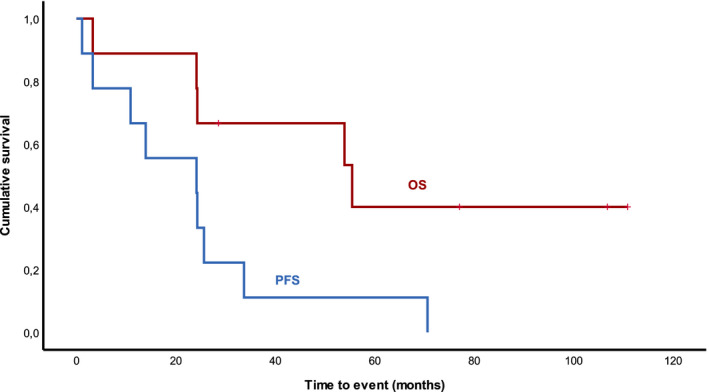
Overall (OS) and progression‐free survival (PFS) in the frontline setting

Median follow‐up was 30 months; median overall survival (OS) and progression‐free survival (PFS) were 24 and 13 months, respectively (Figure 1). Median time to next treatment was 13.5 months. For the patients treated in the frontline setting (nine patients), with a median follow‐up of 54 months, median OS and PFS were 54 months and 14 months, respectively (Figure 2). For the patients treated in the relapsed/refractory setting (28 patients), with a median follow‐up of 28 months, median OS and PFS were 22 months and 11.5 months, respectively. Nevertheless, differences between these data did not reach statistical significance (OS log rank test *p =* 0.23 and PFS log rank test *p =* 0.57).

**FIGURE 3:**
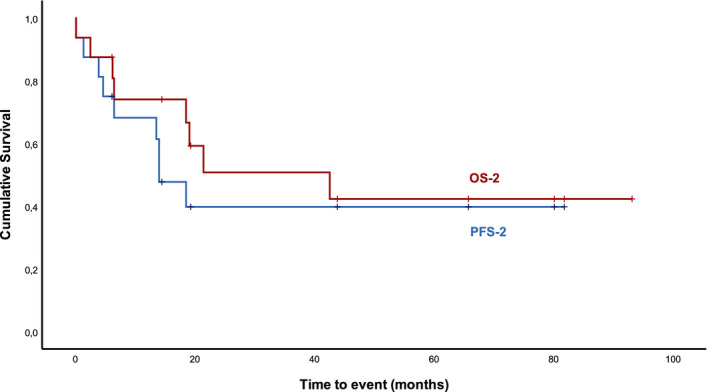
Overall (OS‐2) and progression‐free survival‐2 (PFS‐2) after next line of treatment

Twenty‐four patients relapsed after R‐HDMP. Of these, 18 (75%) patients had been treated on a relapsed/refractory setting and six patients had been treated on frontline setting. Sixteen patients out of the 24 (66.7%) were treated with another line of therapy after R‐HDMP. Median age of this subgroup was significantly lower (72 years old, *p* = 0.04). No patient was retreated with R‐HDMP. Of note, 13 out of these 24 patients (54.2%) had its’ performance status improved at the time of second treatment after R‐HDMP and were subsequently considered fit for chemotherapy or chemo‐immunotherapy. Three other patients were treated with an anti‐CD52 monoclonal antibody or with BTKi. ORR to next line of treatment in this population was 62.6% (CR = 25%, CRi = 6.3%, PR = 31.3%), with 12.5% of SD and 18.8% of progressive disease. One patient was not evaluated. With a median follow‐up of 19.2 months, median OS‐2 and PFS‐2 were 21.4 and 13.5 months, respectively (Figure 3).

**FIGURE 4:**
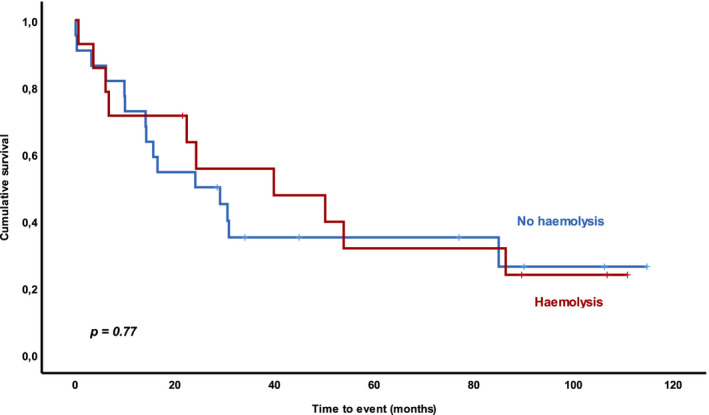
Overall survival (OS) according to the presence of autoimmune haemolytic anaemia

As mentioned before, we also treated 14 patients with AIHA associated with progressive CLL, with an ORR of 85.7% (CR = 14.3%, PR = 71.4%, PD = 14.3%), obtaining a negative direct antiglobulin test after treatment in 43% of patients. Presence of AIHA was not a significant predictor of shorter OS or PFS (median OS 24 months vs. 24.1 months, respectively, *p* = 0.77; median PFS 10.8 months vs. 13.1 months, respectively, *p = *0.67) (Figure 4).

Of the four patients with del(17p), none of them received another line of therapy after R‐HDMP. All of them were dead at last follow‐up: two of them died in complete remission and the other two died of progressive disease. Time to death (TTD) ranged from 10 to 85 months.

RAI stage III/IV, age, IgHV mutational status, presence of del(17p), del(13q) or del(11q), CrCl <60 ml/min/1.73 m^2^ and relapsed/refractory disease were not significant predictors of shorter OS or PFS, by univariate analysis. Also, haemoglobin, platelet count, albumin, LDH and CRP levels at time of treatment were not predictors of shorter PFS or OS. Higher level of β2‐microglobulin was a predictor of shorter PFS (*p* = 0.024, HR 1.36, 95% CI 1.042–1.78) and OS (*p* = 0.018, HR 1.63, 95% CI 1.087–2.46) by cox regression univariate analysis.

Twenty‐eight (71.8%) patients died. Of those, 21 (75%) were being treated on a relapsed/refractory setting. Ten patients (35.7%) died due to disease progression with a median TTD of 12 months (IQR: 2.8–24.5 months). Another 11 (39.3%) patients died due to CLL‐unrelated causes with a median TTD of 40 months (IQR: 14.2–57.2 months). Seven other patients (25%) died due to infection and, of these, two died during treatment, revealing a treatment‐related mortality of 7%, while the other five had a median TTD of 15.7 months (IQR: 5–40). Eleven patients were still alive at the last follow‐up analysis (January of 2019). Of those 11 patients still alive, seven were treated on a relapsed/refractory setting, and eight out of the 11 patients proceeded to another line of therapy after R‐HDMP.

## 4 CONCLUSIONS/DISCUSSION

With R‐HDMP protocol, we treated a cohort of patients with highly unfavourable characteristics: median age of 77 years, more than 50% with cardiovascular disease and 30% with KDIGO stage 3 chronic kidney disease. Around 20% of patients had severe anaemia or thrombocytopenia at the time of treatment. Despite this profile, most patients completed the treatment plan, unfortunately with two deaths during treatment to declare. With this protocol, we achieved an ORR of 64%, similar in patients in frontline and relapsed/refractory setting. Also, this protocol allowed for a rapid disease control with median time for normal lymphocyte and haemoglobin count of less than 30 days. Surprisingly, thrombocytopenia was far more persistent after R‐HDMP than other cytopenias. Median OS reached 24 months and median PFS 13 months in a group of elderly, frail patients with numerous and severe comorbidities, with an unfavourable cytogenetic profile. The toxicity profile was acceptable with only infectious and haematologic grade 3–4 adverse events to declare. Around 30% had grade 3–4 infectious adverse events and around 15% had grade 3–4 cytopenias. This protocol also showed good efficacy in both frontline and relapsed/refractory setting, with an advantage on median OS of those treated in the frontline setting, albeit without statistical significance. Despite the small subgroup analysis, patients with del(17p) also achieved a good response, in accordance with previous publications.^17^

After R‐HDMP, more than half of the patients who relapsed were able to proceed to a next line of therapy, despite their advanced age and severe comorbidities. Most of them had their performance status improved at the time of next line of treatment, and were treated with chemotherapy or chemo‐immunotherapy protocols. In this setting, patients lived for a median of 21 months, 13 of them without relapse, with a median time to relapse of 13 months, which are very interesting results especially considering their baseline clinical and cytogenetic characteristics. In our sample, AIHA was not associated with shorter OS or PFS, in agreement with other studies.^6^ Once again this highlights the importance of not considering AIHA as a negative prognostic factor.

In fact, the subgroup of patients with AIHA had a better ORR after R‐HDMP (ORR 85.7% vs. ORR 54.5%) with 43% of the patients achieving a negative direct antiglobulin test after treatment. This treatment proved to be a useful tool in controlling complex AIHA. This means that along with disease control, anaemia resolution was attained in most patients. On the other hand, these data may suggest that R‐HDMP can also be part of the treatment strategy of young and fit patients when a quick resolution of autoimmune and symptomatic anaemia is needed. Its rapid and effective disease control, as well as the rapid control of the autoimmune cytopenias, may lead to significant improvement in performance status. An improvement in performance status is many times a key factor to proceed to a more intensive regimen, allowing the patient to achieve a longer and deeper response. In this context, R‐HDMP may also work as a bridge to more intensive treatment protocols, when autoimmune cytopenias are a limiting factor.

Twenty‐eight patients eventually died and most of them were being treated on a relapsed/refractory setting. Nevertheless, 39% of patients died due to CLL‐unrelated causes, with median TTD of 40 months.

Our results were inferior to other similar studies, where ORR reached 96%,^17^ 75%,^14^ and 78%.^16^ However, Castro et al addressed only patients in the frontline setting, while most of our patients had relapsed/refractory disease. On the other hand, Šimkovič et al opted for a higher number of cycles (total of 8 cycles) and Bowen et al designed a protocol with a higher dose of steroids (methylprednisolone 1 g/m^2^ for 5 days). These details may justify the higher ORR but also some of the adverse events mentioned in these studies: steroid‐induced psychosis, severe metabolic decompensation in diabetic patients and the need for inpatient administration of the first cycle to prevent tumour lysis syndrome and severe acute kidney injury. Importantly, we did not observe any of these adverse events in our study. On the other hand, our rate of grade 3–4 infectious complications during treatment was higher than in some of these studies. We documented 31% of grade 3–4 infections while Castro et al^17^ refer only 17%. However, Bowen et al^16^ refer 29% of infectious adverse events, 14% of them grade 5. Similarly to our study, Šimkovič et al^14^ report 27% of grade 3–4 infectious adverse events, even though they opted for a higher number of cycles. Of note, patient median age in these studies was 65, 66 and 67 years old, respectively, approximately 10 years younger than in our cohort, which argues in favour of our toxicity profile.

In 2019, Pileckyte et al^15^ published their results on an old and frail population (median age of 73 years old) showing an ORR of only 28%, all PR. However, they showed a similar median PFS of 11 months and a greater median OS of 68 months, with a median follow‐up of 50 months. Their protocol aimed for 3 days of steroids (methylprednisolone 1 g/m^2^) and a higher dose of rituximab (1000 mg/m^2^). They only report 16% of grade 3–4 infectious adverse events and no deaths during treatment, in a patient cohort with characteristics similar to ours’.

On the specific setting of CLL plus AIHA, our results were similar to those presented by Mauro et al. This author presents 52 patients with CLL and AIHA who were treated either with steroids or with steroids plus chlorambucil, attaining 84% of response. The median age of their cohort was 69 years. Most patients (48) were treated with prednisolone and chlorambucil and only 4 were treated with prednisolone. However, they report a median time to relapse of 19 months, larger than in our cohort.^19^

R‐HDMP protocol is not a revolutionary option for CLL treatment, especially in an era where BTKi and Bcl‐2 inhibitors have entered broad clinical practice. However, one question remains: what is the best treatment for elderly, fragile patients, considering that most of them have severe comorbidities and are taking multiple medication? The burden of polypharmacy is thought to have many different dimensions, from an increased risk of serious adverse events, to dangerous drug interactions, lack of adherence and greater health care costs.^9^ The economic advantage of BTKi has been proven even in the relapsed/refractory setting.^20^ However, strict adherence to therapeutic schedule has been associated with longer survival and those who do not follow it strictly tend to be older.^21^ A relevant topic is the report of 36% grade 3–4 infectious adverse events in patients with R/R CLL treated with a BTKi, despite being younger than the patients from our study.^22^ On the other hand, long‐termed fixed therapy in older and comorbid patients has recently shown only 17.5% of grade 3–4 infections.^10^ Nevertheless, these authors suggest a 2‐year long treatment protocol.

R‐HDMP is not similar to targeted therapies in terms of efficacy, which means it cannot be presented as an alternative. Nevertheless, we find this protocol to be useful in this very specific subset of older and frail patients, concerning not only efficacy, but also tolerability, adherence to therapy and drug interactions. Also, in the current pandemic era, one might find an oral‐targeted therapy a considerable advantage comparing to an intravenous treatment. However, our daily practice let us know that patients on oral‐targeted therapies have to run through a period of very close observation, sometimes with twice a week consults. On this specific topic, a short duration treatment protocol, even if at in‐hospital setting may be an advantage to consider. Moreover, R‐HDMP is administered independently of patient adhesion to the treatment regimen, which is also an advantage on a cohort of elderly patients. Prospective studies would be needed to answer these specific questions in such a specific setting.

This study has some limitations as any retrospective analysis. The small number of patients allowed us to draw conclusions about our sample but makes it impossible to extrapolate the results to all CLL patients, and we recognise that the reproducibility of these data may be limited. In our analysis we included patients both in frontline and relapsed/refractory setting, and despite ORR and survival analysis was not significantly different, those data should be interpreted with caution. Also, the small number of patients may have biased the analysis of response and survival predictors, explaining why the well‐known negative prognostic factors (such as high‐risk disease, del17p, increasing age, unmutated IgHV gene) failed to predict worse outcomes in our sample. The heterogeneity of previous treatments as well as the heterogeneity of therapies administered after R‐HDMP may also have influenced the outcomes evaluated in this study. Besides, patient supportive care may also have differed from one physician to another, considering the antifungal and antiviral prophylaxis regimen, transfusion thresholds, and the decision for inpatient care. Nevertheless, these are real‐life results from real‐life patients above 70 years old, with end‐organ damage and severe comorbidities, who also need treatment and generally are not included in clinical trials.

In conclusion, based on our results, R‐HDMP may be useful in some particular clinical settings, where a rapid disease control is warranted in elderly and frail patients. We highlight 30% of severe infections, but low risk of severe myelotoxicity and other severe adverse events. It was shown to work as a bridge to other lines of treatment, including chemo‐immunotherapy. In an era of new drugs administered for long periods of time or even until progression, with a significant economic burden, drug interactions and adherence issues, this protocol offers a time‐limited out‐patient therapy, with few drug interactions. However, durable responses are rare, so further optimisations of this protocol should be attempted.

## CONFLICT OF INTEREST

Ana Vagos Mata has no conflict of interest to declare. Eduardo Espada has received consulting fees from MSD and honoraria form MSD, Pfizer and Roche. Daniela Alves has received occasional consulting fees for Janssen Cilag, Abbvie, Roche, Takeda, AstraZeneca and occasional speaker fees for Janssen Cilag, Abbvie, Roche, Takeda, Gilead Sciences. Blanca Polo has no conflict of interest to declare. Maria João Costa has no conflict of interest to declare. Conceição Lopes has no conflict of interest to declare. João F. Lacerda has no conflict of interest to declare. João Raposo has received occasional consulting fees for Janssen Cilag, Abbvie, Takeda, Merck Sharp & Dome, Gilead Sciences, AstraZeneca.

## ETHICAL STATEMENT

This study was conducted according to Helsinki declaration principles and under the approval of local Ethics Committee. All patients signed an informed consent before treatment, according to local practice.

## Data Availability

The authors state every information on this manuscript is true and allow for data sharing. The data that support the findings of this study are available from the corresponding author upon reasonable request.
